# Supplementary figures and images for: Recombinant biosensors for multiplex and super-resolution imaging of phosphoinositides
Source: J Cell Biol. 2024 Apr 5;223(6):e202310095. doi: 10.1083/jcb.202310095 (PMC10996583; doi:10.1083/jcb.202310095)

PIP probe:

PI  
PI(3)P  
PI(4)P  
PI(5)P  
PI(3,4)P<sub>2</sub>  
PI(3,5)P<sub>2</sub>  
PI(4,5)P<sub>2</sub>  
PI(3,4,5)P<sub>3</sub>

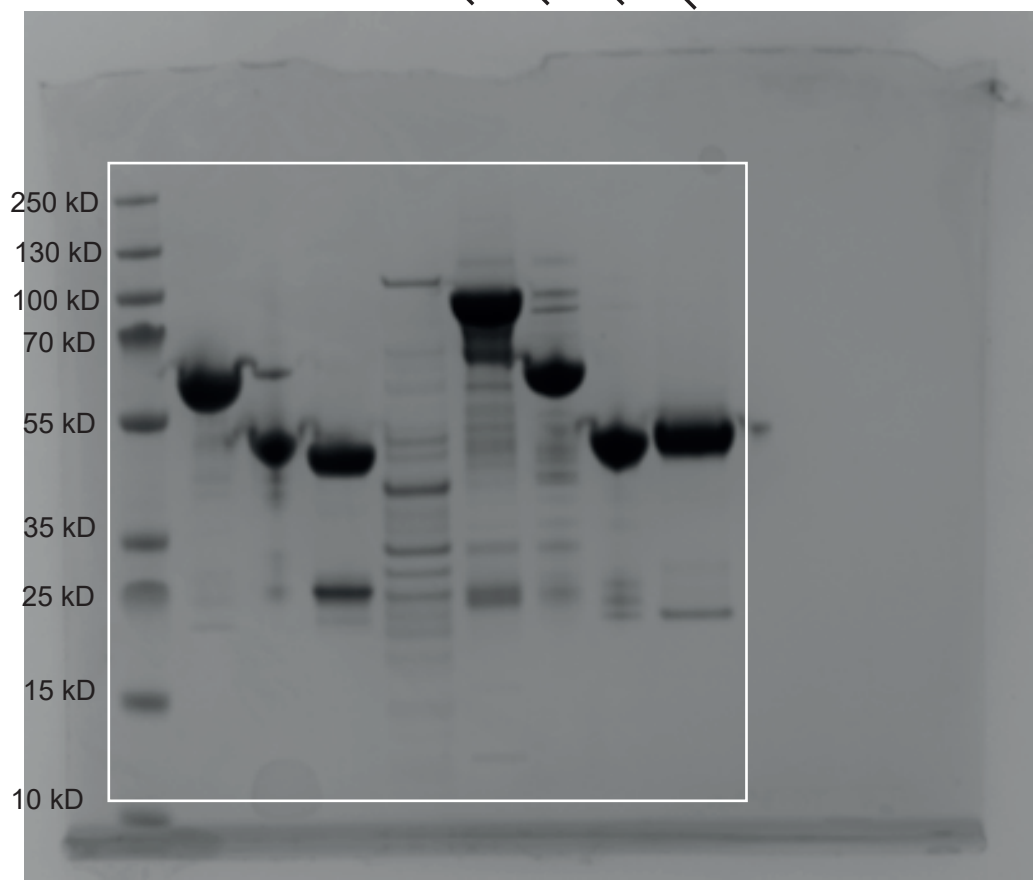

Supplement: SourceData F1 — is the source file for Fig. 1. [file JCB_202310095_SourceDataF1.pdf]

6xHis-SNAP-PLC $\delta$ 1  
Imidazole

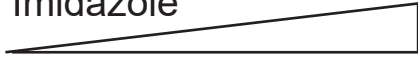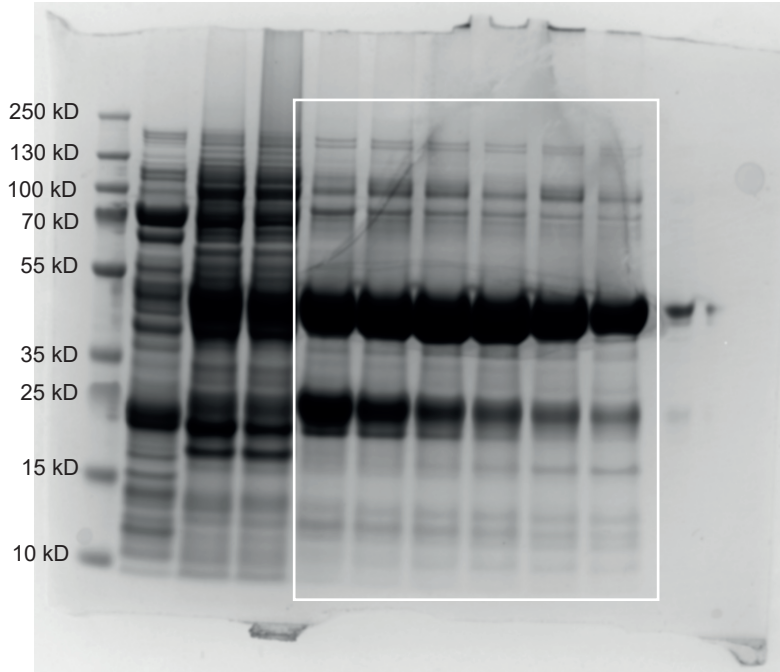

Supplement: SourceData FS3 — is the source file for Fig. S3. [file JCB_202310095_SourceDataFS3.pdf]
